# Eco-physiological response of *Phaeocystis antarctica* and *Fragilariopsis* sp. to increases in irradiance and temperature

**DOI:** 10.1093/plankt/fbaf023

**Published:** 2025-06-15

**Authors:** Antonia Cristi, Stacy Deppeler, Alexia Saint-Macary, Andrew Marriner, Mikel Latasa, Cliff S Law, Andrés Gutiérrez-Rodríguez

**Affiliations:** National Institute of Water and Atmospheric Research, 301 Evans Bay Parade, Hataitai, Wellington 6021, New Zealand; Department of Marine Science, University of Otago, 310 Castle Street, Dunedin 9016, New Zealand; National Institute of Water and Atmospheric Research, 301 Evans Bay Parade, Hataitai, Wellington 6021, New Zealand; National Institute of Water and Atmospheric Research, 301 Evans Bay Parade, Hataitai, Wellington 6021, New Zealand; Department of Marine Science, University of Otago, 310 Castle Street, Dunedin 9016, New Zealand; National Institute of Water and Atmospheric Research, 301 Evans Bay Parade, Hataitai, Wellington 6021, New Zealand; Instituto Español de Oceanografía, Centro Oceanográfico de Gijón/Xixón (IEO,CSIC), Av. del Príncipe de Asturias, 70 Bis, 33212 Gijón, Asturias, Spain; National Institute of Water and Atmospheric Research, 301 Evans Bay Parade, Hataitai, Wellington 6021, New Zealand; Department of Marine Science, University of Otago, 310 Castle Street, Dunedin 9016, New Zealand; National Institute of Water and Atmospheric Research, 301 Evans Bay Parade, Hataitai, Wellington 6021, New Zealand; Instituto Español de Oceanografía, Centro Oceanográfico de Gijón/Xixón (IEO,CSIC), Av. del Príncipe de Asturias, 70 Bis, 33212 Gijón, Asturias, Spain

**Keywords:** Fragilariopsis, *Phaeocystis antarctica*, temperature, irradiance, chemostats

## Abstract

*Phaeocystis antarctica* and *Fragilariopsis* are key phytoplankton taxa in the Southern Ocean that have different bloom magnitude and phenology, reflecting their differing physiological traits. Here, we investigate the physiological response of *Fragilariopsis* sp. and colony-forming *P. antarctica* to warmer and high irradiance conditions using chemostat experiments under low light/low temperature (LL/LT) and high light/high temperature (HL/HT). C:N and C:Chl*a* ratios increased under HL/HT in both species, whereas the *Fragilariopsis* sp. Si:C ratio showed no significant variation between treatments despite Si:N being 1.4-fold higher under HL/HT. The *P. antarctica* colony to a single-cell ratio exhibited a 2.3-fold increase under HL/HT but with no change in the size of individual cells. On the contrary, *Fragilariopsis* sp. cell size decreased 1.3-fold without affecting cellular silica content. DMSPt:C and DMS:C increased in both species under HL/HT with no effect of treatment on DMSPt:DMS for either species. Primary pigment markers for taxonomic identification were unaffected by treatment, but pigments in the xanthophyll cycle increased under HL/HT with higher concentrations in *Fragilariopsis* sp. and a higher rate of epoxidation in *P. antarctica.* Results indicate a greater tolerance in *P. antarctica* to increased irradiance and warming than previously described, suggesting that it may be competitive with *Fragilariopsis* sp. under conditions usually associated with diatom dominance.

## INTRODUCTION

Bacillariophyta (diatoms) and Prymnesiophyceae (*P. antarctica*) are some of the main bloom-forming taxa in the Southern Ocean, with differing eco-physiological characteristics that determine the timing of their proliferation. The accumulation of the colonial form of *P. antarctica* has been previously attributed to tolerance of low and variable irradiance associated with deeper surface mixed layers (Arrigo *et al.,* 1998). Diatoms subsequently bloom under more stratified conditions (Arrigo *et al.*, 1999), reflecting their greater tolerance at higher irradiance (Arrigo *et al.,* 2010). However, bloom dynamics in the Southern Ocean are changing in magnitude, seasonality and phenology in response to climate change (Thomalla *et al.,* 2023). Future warmer conditions projected for the end of the century may result in increases in stratification and shallower mixed layer depth (Rickard and Behrens, 2016; Xue *et al.,* 2024), affecting photosynthetic efficiency via increased exposure to higher light levels (Bopp *et al.,* 2001; Boyd *et al.,* 2008) and reduced inputs of iron from subsurface waters (Smith *et al.,* 2012). Based on previous experiments (Xu *et al.,* 2014; Zhu *et al.,* 2017), it is anticipated that these climate change–driven alterations may benefit diatoms relative to *P. antarctica*. Conversely, field observations have reported a low correlation between mixed layer depth and the abundance of both diatoms and *P. antarctica* (Cristi *et al.,* 2024) and also *P. antarctica* dominance in shallower mixed layers (Mangoni *et al.,* 2019), suggesting that *P. antarctica* may be more competitive than previously reported.

Warmer temperatures are expected to affect phytoplankton physiology differently depending on the thermal tolerance of each species (Bishop *et al.,* 2022) and ultimately determine species biogeography and phenology of blooms (Thomas *et al.*, 2012). Temperature also influences tolerance to light by affecting light-harvesting pigment cell concentrations (Finkel *et al.,* 2010) and also iron metabolism with higher temperatures decreasing tolerance of iron limitation (Sunda and Huntsman, 2011). Ultimately, this may have implications for *Phaeocystis* colony formation (Wang *et al.,* 2010), *Fragilariopsis* sp. silica cell concentration (Xu *et al.,* 2014) and more generally for cell size (Atkinson *et al.,* 2003).

The effect of increasing irradiance due to shallower mixed layers on bloom dynamics will depend on species-specific mechanisms for optimizing physiological performance under prevailing light regimes. One of the main mechanisms underpinning photoacclimation is the xanthophyll cycle (Goss and Jakob, 2010), which is carried out by the pigments diadinoxanthin (Ddx) and diatoxanthin (Dtx) in both diatoms and Prymnesiophyceae (Strain *et al.,* 1944). During the xanthophyll cycle, Ddx is transformed into Dtx by a de-epoxidation process activated by light (Goss and Jakob, 2010). Culture experiments conducted with single-cell *P. antarctica* have shown that this species can only sustain the xanthophyll cycle for short periods and instead relies more on photo-repair mechanisms (Kropuenske *et al.,* 2009). This makes *P. antarctica* more susceptible to photoinhibition than *Fragilariopsis* sp., which can sustain the activation of the xanthophyll cycle at high irradiance for long periods, hence reducing photodamage associated with high light exposure (Kropuenske *et al.,* 2010).

Any alteration in bloom dynamics between diatoms and *P. antarctica* in response to climate change may have implications for food webs and carbon fluxes. Zooplankton graze more effectively on diatoms than *P. antarctica* colonies, which supports a more efficient transfer of carbon to higher trophic levels (Schnack-Schiel and Isla, 2005). Diatoms are also considered a high-export group (Smetacek, 1999), although their contribution differs amongst species depending on cell size and density, with larger and denser cells sinking faster (Raven and Waite, 2004; Finkel and Kotrc, 2010). In the case of *P. antarctica*, early studies attributed a high export potential through sinking associated with low remineralization in the upper water column (DiTullio *et al.,* 2000) but recent studies have pointed towards deep mixing rather than sinking as the reason for observed export events involving *P. antarctica* (Jones and Smith, 2017). *Phaeocystis antarctica* also plays a significant role in sulphur cycling due to its high intracellular dimethylsulfoniopropionate (DMSP) content, which may subsequently influence aerosol and cloud formation via the production and emission of dimethylsulfide (DMS) (Liss *et al.,* 1994). At a cellular level, DMSP may act as an antioxidant (Sunda *et al.,* 2002), osmoprotectant (Lyon *et al.,* 2016) and a cryoprotectant (Nishiguchi and Somero, 1992) and also has a potential role as an info-chemical (Seymour *et al.*, 2010), as a grazing attractant (Shemi *et al.,* 2021) or a deterrent (Fredrickson and Strom, 2009).

To date, most experimental studies investigating the influence of environmental drivers on the relative dominance of these phytoplankton groups have compared the response of diatoms and single-celled *P. antarctica* (Zhu *et al.,* 2016; Trimborn *et al.,* 2017, 2019; Beszteri *et al.,* 2018), or not specified morphotype (Kropuenske *et al.,* 2009; Arrigo *et al.,* 2010), with only two studies examining colony-forming strains (Xu *et al.,* 2014; Zhu *et al.,* 2017). Experiments using natural communities have also explored the response of these two groups to various physicochemical factors (Feng *et al.,* 2010). Here, we conducted two chemostat experiments using monospecific cultures of *Fragilariopsis* sp. and colony-forming *P. antarctica,* under two combinations of irradiance and temperature to test the physiological and ecological effects of projected future warming (+2°C) and increased light on each species. Specifically, this study focuses on the acclimatory response of (i) cell biomass and elemental composition, (ii) photophysiology and (iii) DMSP and DMS production.

## MATERIALS AND METHODS

### Experimental framework

Two independent chemostat experiments were conducted under controlled light, macronutrient and temperature conditions using a Biostat® B benchtop bioreactor with twin double wall Univessel® Glass 2 L culture vessels (Sartorius stedim biotech, Göttingen, Germany), hereafter referred to as the chemostat (Supplementary Fig. 1). A detailed explanation of the setup is provided in the supplementary material (S1.1). Chemostat systems allow the maintenance of a stable physiology by balancing cell growth with a constant rate of nutrient supply (Ziv *et al.,* 2013). This is achieved by establishing a constant inflow of fresh media balanced by an outflow that dilutes the culture at a constant rate (i.e. dilution rate). After several generations, cells attain balanced growth—in relation to an acclimated nutrient limitation (in this case, nitrogen), indicating that cell metabolic demands are met and reflected by maintaining a stable biomass (Parkhill *et al.,* 2001)—which can be maintained for long periods. Under these steady-state conditions, the cell growth is acclimated to nutrient availability and the cell’s physiology responds exclusively to the treatment (Sciandra *et al.,* 1997), reducing the uncertainties associated with changing biomass and growth conditions inherent to batch cultures (Gresham and Dunham, 2014).

Here, each experiment was performed independently using two phytoplankton strains isolated from Antarctica; the diatom *Fragilariopsis* sp. (single-celled only, RCC6062, isolated from the Ross Sea), and the Prymnesiophyceae *P. antarctica* (colonies and single cells, RCC4024, isolated from West Antarctic Peninsula), supplied by the Roscoff Culture Collection (RCC, Roscoff, France). In the case of *Fragilariopsis* sp., Blastn analysis indicates that the best match is *Fragilariopsis cylindrus* (Supplementary Table I), but we refer to this as *Fragilariopsis* sp. throughout the text for consistency with the RCC database.

Cultures were grown using nitrogen-limited K + Si media for *Fragilariopsis* sp. (20-fold lower concentration relative to Roscoff recommended media strength: 44 μM of NO_3_ and 2.5 μM of NH_4_) and nitrogen-limited K/2ET for *P. antarctica* (8-fold lower nitrogen concentration: 35 μM NO_3_ and 0.5 μM NH_4_) (https://roscoff-culture-collection.org/culture-media). The differences in nitrogen limitation between experiments were determined by the tolerance to nitrogen deficit of each species, with *P. antarctica* unable to grow at 20-fold nitrogen limitation. Although iron is the main limiting nutrient in the Southern Ocean (Martin *et al.,* 1990), the chemostat does not allow for an iron-clean experiment. Therefore, iron was not limited and, in both cases, was supplied at the concentration specified in the media. Phosphorus concentration remained at the concentration suggested by RCC (10 μM for *Fragilariopsis* sp. and 18 μM for *P. antarctica*).

The experiment aimed to test the physiological response to increases of +2°C and light in response to a shallower surface mixed layer within each species. The temperature in Southern Ocean surface waters ranges between −2°C and 10°C, depending on the region (Auger *et al.,* 2021). As the strains used in the experiments were long-term acclimated to 4°C, we used this as the control temperature, as with previous experiments on Southern Ocean phytoplankton (Stefels and Van Leeuwe, 1998; Van Leeuwe *et al.,* 2014), acknowledging that this is higher than surface water temperatures in more southerly waters around Antarctica. Consequently, the low-light and low-temperature conditions (LL/LT) were set at 4°C and 25-μmol m^−2^ s^−1^ of photosynthetic photon flux density (PPFD). The “treatment” representing a future scenario of increased temperature and light was then set at +2°C higher (+6°C) and 125-μmol m^−2^ s^−1^ PPFD to simulate high light and high temperature (HL/HT). Light levels were chosen based on low and high irradiance levels used in previous experiments (Arrigo *et al.,* 2010; Xu *et al.,* 2014). In both cases, a constant irradiance was used to simulate Antarctic summer daylight conditions.

**Fig. 1:**
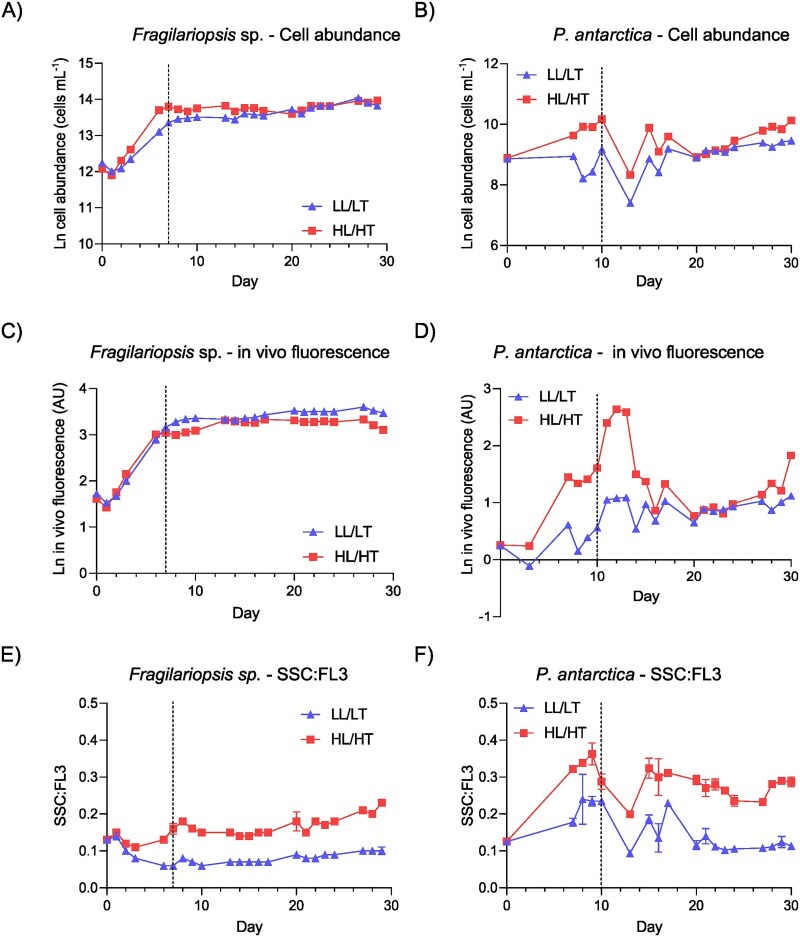
Experiment progression of growth and fluorescence in *Fragilariopsis* sp. (left) and *P. antarctica* (right). Plots (**A**) and (**B**) are cell abundance measured by flow cytometry; (**C**) and (**D**) *in vivo* fluorescence expressed in arbitrary units (AUs); and (**E**) and (**F**) SSC:FL3 ratio as a measure of fluorescence per cell (single cells only). Dashed lines indicate the day each chamber was connected to the continuous exchange of culture and media. Note that the *y*-axis scale differs with the parameter. LL/LT = low light/low temperature. HL/HT = high light/high temperature.

The initial inoculum was prepared in triplicate batch cultures and grown for 25 days in the same nutrient-limited media as prepared for each chemostat experiment at LL/LT conditions. Batch cultures were then pooled together in a single sterile bottle and gently mixed before ~150 mL was inoculated into each chemostat chamber, previously filled with 1.5 L of sterile media. Chambers were then topped up to 2 L with fresh media and left overnight without stirring. For the *Fragilariopsis* sp. experiment, the cultures were stirred at 20 rpm for the remainder of the experiment. There was no stirring in the *P. antarctica* experiment, except for the 2 hours prior to daily sampling, to minimize potential damage to colonies. Cultures were grown in the chemostat chambers under the specified conditions for 7 days in the case of *Fragilariopsis* sp. and 10 days for *P. antarctica,* before connecting to a continuous exchange of culture and media using two independent peristaltic pumps that controlled the inflow and outflow rates. In chemostat systems, the growth rate is set by the dilution rate (Laws *et al.,* 1995), which were 0.15 and 0.10 L day^−1^ for *Fragilariopsis* sp. and *P. antarctica*, respectively, and were less than the maximum growth rates under nitrogen-limited media determined previously at LL/LT conditions (μmax *Fragilariopsis* sp*.*: 0.2 L day^−1^; μmax *P. antarctica*: 0.12 L day^−1^). Experiments were maintained at constant temperature, irradiance and dilution rate conditions for 3 weeks before the final sampling. For each experiment, both chambers were harvested in the same 24-hour period: Day 29 for *Fragilariopsis* sp. and Day 30 for *P. antarctica* (Fig. 1).

### *In vivo* fluorescence and flow cytometry measurements

*In vivo* fluorescence and flow cytometry (FCM) cell counts were used to assess population biomass dynamics throughout the experiment (Fig. 1). For *in vivo* fluorescence, 4 mL from each culture chamber were collected in borosilicate glass tubes, gently mixed in the dark and immediately read three times using a 10 AU Field fluorometer (Turner designs, CA, USA). FCM samples were collected following Marie *et al.* (2014) with 1.5 mL of culture preserved with 15 μL of a mixture of glutaraldehyde (25%, Sigma-Aldrich, Missouri, US) and pluronic acid (Poloxamer 188 solution 10%, Merck, New Jersey, US) and incubated in the dark for 15 minutes at room temperature. Samples were then flash-frozen in liquid nitrogen, stored at −80°C and subsequently analysed using a BD Accuri C6 Plus (BD Biosciences) flow cytometer following standard procedures (Tarran and Bruun, 2015). The ratio between chlorophyll-*a* (Chl*a*) fluorescence (FL3) and side scatter (SSC) (FL3:SSC) was used as a proxy of cell pigment content for characterizing photoacclimation on single cells for both species (Gutiérrez-Rodríguez *et al.,* 2011). Bacterial cell counts were determined using SYBR Green II [10.000X concentrate in Dimethyl sulfoxide (DMSO), ThermoFisher Scientific, MA, USA]. Samples were incubated in the dark for 15 minutes before analysis using the same flow cytometry specified above, using the SSC and FL1 plot.

### Particulate organic matter, total Chl*a* and biogenic silica

Particulate organic carbon (POC) and nitrogen (PON) were collected by filtering 50 mL of culture on a pre-combusted 25 mm 0.7 μm pore size glass fibre filter (Whatman GF/F). Blanks were collected by placing pre-combusted Whatman GF/F filters on the same filtration rack without filtering water. Filters were acidified by fumigation over concentrated hydrochloric acid in a glass desiccator with Teflon-lined shelf, followed by air-drying in a fume hood for 2 hours. Analysis was conducted using a DELTA V Plus continuous flow isotope ratio mass spectrometer linked to a Flash 2000 elemental analyser using a MAS 200 R autosampler (Thermo-Fisher Scientific, Bremen, Germany). POC and PON from blanks were subtracted in culture samples. Samples for total Chl*a* (TChl*a*) were collected by filtering 50 mL of culture through Whatman GF/F filters, dry-blotted, folded, wrapped in aluminium foil and stored at −80°C. Analysis was conducted using a Turner Designs 10 AU field fluorometer following 90% acetone extraction and standard fluorometric technique after Strickland and Parsons (1972). Chl*a* cell content is only available for *Fragilariopsis* sp. due to its single-celled form and was determined by dividing the Chl*a* concentration by the number of cells estimated by FCM. An additional 20 mL of culture was sampled for the determination of biogenic silica (bSi) in the *Fragilariopsis* sp. experiment. Samples were filtered through 0.6 μm pore size polycarbonate filters (Merck Millipore, MA, US) and stored at room temperature until analysis, following procedures described in Ragueneau and Tréguer (1994).

### Carbon biomass and cell size for *Fragilariopsis* sp*.*

POC concentration was used to estimate cell carbon content. To determine if it was appropriate to use POC as a proxy for carbon for this species, bacteria cell counts were transformed into carbon and nitrogen using the cell carbon content of “Oceanic, Southern (65°S)” from Fukuda *et al.* (1998), which determined a low contribution of bacteria to the POC (1.2% for HL/HT and 0.6% for LL/LT) and PON (0.2% for HL/HT and 0.5% for LL/LT) pools. Since the correlation between POC and bacteria cell counts was not significant (linear regression, *F* = 1 457, *P*-value = 0.2506) (Supplementary Fig. 2), we use POC and PON as a proxy for carbon (C) and nitrogen (N) for this study. In both parameters, the concentration of C and N was divided by phytoplankton cell abundance to estimate C and N cell content. FSC measurements provide an estimate of cell size in arbitrary units (Rieseberg *et al.,* 2001); therefore, relative alterations in size were determined by looking into variations in forward scatter (FSC) and SSC from flow cytometry between treatments (Supplementary Table II) since specific cell size measurements are not available.

### Carbon biomass, cell and colonial size for *P. antarctica*

A combination of size-fractionated Chl*a* (SFChl*a*) that separated >20 μm and <20 μm fractions and light microscopy was used to quantify colonial biomass, size and abundance relative to single cells. For SFChl*a,* 20 mL of sample was filtered under low vacuum through a 20 μm pore size polycarbonate filter to retain large colonies, followed by 0.2 μm filtration to retain the single cells and small colonies passing through the 20 μm filter (Tang *et al.,* 2008). Analysis of SFChl*a* filters was conducted using 90% acetone extraction followed by spectrofluorometer on a Varian Cary Eclipse fluorometer following method APHA 10200 H (American Public Health Association, 2005). For light microscopy, 3 mL of culture from each chamber was collected and preserved with acidic Lugol’s iodine solution (1% final concentration) and stored in the dark at 4°C. Analysis was conducted by settling 1 mL of preserved sample in a Utermöhl sedimentation chamber for 1 hour. All colonies found in the chamber were counted, and their length was measured for size determination using an Olympus IX73 inverted microscope and CellSense software. The length of single cells and those forming colonies (40–50 in each case) were measured to estimate cell carbon content, assuming a spherical shape, as described in Mathot *et al.* (2000), and using the following equation from Eppley *et al.* (1970):


$$ \mathrm{Log}\ {\mathrm{C}}_{\mathrm{cell}}=0.94\ \left(\log{\mathrm{Vol}}_{\mathrm{cell}}\right)-0.6 $$

### High-performance liquid chromatography analysis

High-performance liquid chromatography (HPLC) pigment analysis was conducted by low-vacuum filtration of a 50 mL sample from each chamber through GF/F filters. Pigment extraction was conducted by sonicating the filter in 2.5 mL of 90% acetone and storage at −20°C overnight, with the extract filtered through GF/F. Analysis was carried out using an Agilent HPLC 1200 system equipped with a G1311A quaternary pump, a G2258A autosampler, a G1316B column thermostat and a G1315C diode array detector following Latasa *et al.* (2022). The de-epoxidated state of the xanthophyll cycle (DES)—an estimate of the conversion of Ddx to Dtx—was calculated by dividing the concentration of Dtx by the total xanthophyll pigments (Ddx + Dtx) according to Kropuenske *et al.* (2010).

### Dimethylsulfide and total dimethylsulfoniopropionate

Samples for dimethylsulfide (DMS) were collected by transferring 1 mL of culture from each chamber to a 10 mL syringe previously filled with ultrapure MilliQ water and subsequently injected onto a GF/F filter in a sparging unit. Samples for total dimethylsulfoniopropionate (DMSPt)—which includes particulate and dissolved DMSP plus DMS—were collected in duplicate by pipetting 1 mL of sample into 5 mL glass vials containing 4 mL of MilliQ water and adding two pellets of NaOH. Vials were gas-tight-sealed and stored in the dark at room temperature. Analysis was conducted using a purge and trap preconcentrator followed by separation using an Agilent 6 850 gas chromatograph with detection by an Agilent 355 sulphur chemiluminescence detector (Saint-Macary *et al.,* 2021). DMS(P) is used in the text when generally referring to both DMSPt and DMS. We did not quantify DMS losses to the headspace, but since there was no aeration in the chemostat chambers, this loss is expected to be low.

### Statistical analysis

The chemostat experimental set-up provided two unreplicated treatments, and so subsamples were collected for each parameter as pseudoreplicates to provide an estimate of variability within sampling and analysis. As in similar experiments where replicated treatments were not possible (Deppeler *et al.,* 2018), pseudoreplicates were treated as true replicates to provide an assessment of differences between treatments. This approach was also supported by the chemostat approach in maintaining stable physiological conditions for long periods (Kolber *et al.,* 1988; Laws *et al.,* 1995; Gutiérrez-Rodríguez *et al.,* 2014), thus ensuring that the observed responses were due to treatment and not from growth stages or shock responses (see Experimental framework section).

The treatment effect within each experiment was evaluated using Student’s *t*-test. Differences between different morphotypes (single-celled and colonial) in the *P. antarctica* experiment were determined using a two-way analysis of variance (ANOVA) followed by Tukey’s honest significant difference (HSD) (R package “*Stats”* version 3.6.2). Two-way ANOVA was also used to compare the response between species on pigment concentration and elemental composition. Results from two-way ANOVA and Tukey’s HSD test can be found in Supplementary Tables III and IV.

## RESULTS

### Elemental composition

**Fig. 2:**
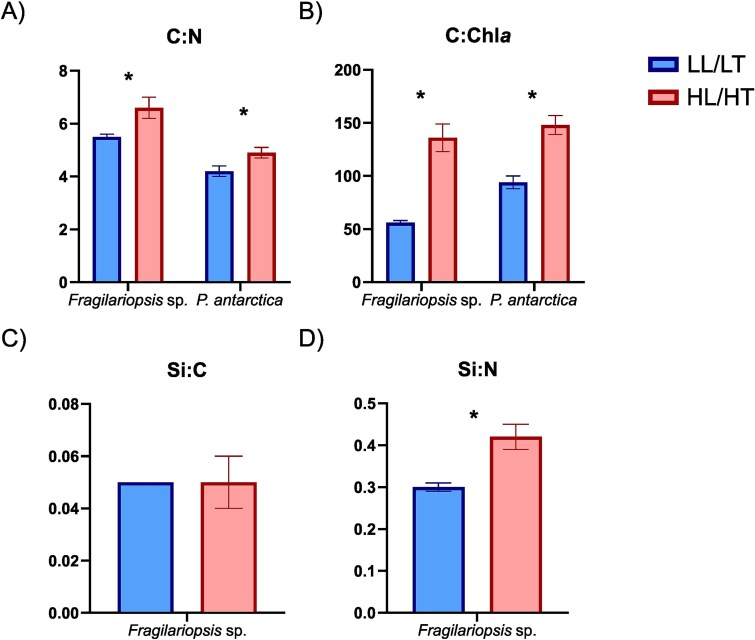
Stoichiometry ratios for each species and treatment. (**A**) C:N, (**B**) C:Chl*a*, (**C**) Si:C and (**D**) Si:N. The colour blue indicates low light/low temperature (LL/LT) and red high light/high temperature (HL/HT). *t*-test was used for statistical analysis of differences between treatments, *P* < 0.05 are indicated with an asterisk (^*^). Silica ratios are only available for *Fragilariopsis* sp.

Bulk C:N and C:Chl*a* ratios were higher under HL/HT for both species (Fig. 2). The C:Chl*a* ratio exhibited a greater increase in *Fragilariopsis* sp*.* under HL/HT (2.4-fold; *t*-test, *t* = 12.33, df = 11.203, *P* < 0.001) than *P. antarctica* (1.6-fold; *t*-test, *t* = 16.95, df = 14.511, *P* < 0.001). In the case of C:N, both species had a similar increase under HL/HT (1.2-fold), which maintained the higher C:N ratio observed in *Fragilariopsis* sp. under LL/LT (1.3-fold higher than *P. antarctica*). The *Fragilariopsis* sp. Si:C ratio showed no significant variation between treatments *(t*-test, *t* = 0.862, df = 17.27, *P* = 0.401), although Si:N was 1.4-fold higher under HL/HT *(t*-test, *t* = 18.88, df = 21.45, *P* < 0.001) (Supplementary Table V). As POC was unaffected by treatment, the stoichiometric changes observed in *Fragilariopsis* sp. were due to variations in PON, TChl*a* and Si, whereas *P. antarctica* had significant variations in POC and TChl*a* but not in PON concentration (Table I).

**Table I:** Final biomass for each species and treatment

Treatment	POC	PON	TChl*a*	bSi
	(μg L^−1^)	(μg L^−1^)	(μg L^−1^)	(μg L^−1^)
*Fragilariopsis* sp.				
LL/LT	4 187 ± 86	766 ± 8	80.5 ± 12. 7	9.96E-4 ± 4.0E-5
HL/HT	4 103 ± 166	623 ± 30	34.2 ± 8.4	1.11E-3 ± 4.1E-5
* P*-value	0.466	<0.001^*^	<0.001^*^	0.004^*^
* t* value	−0.80	−9.32	−6.10	4.55
df	4.50	3.43	5.20	5.99
*P. antarctica*				
LL/LT	1 211 ± 34	290 ± 13	12.9 ± 0.8	NA
HL/HT	1 532 ± 68	315 ± 7	10.4 ± 0.4	NA
* P*-value	0.006^*^	0.057	0.015^*^	NA
* t* value	7.32	3.02	−4.98	
df	2.94	2.99	3.1	

*t*-test was used for statistical analysis of differences between treatments; *P* < 0.05 are indicated with an asterisk (^*^). LL/LT = low light/low temperature. HL/HT = high light/high temperature.

### Cell size and cellular content

Chl*a* cell content and FL3:SSC were lower under HL/HT in *Fragilariopsis* sp. (Table II). Variations in FSC values between treatments in *Fragilariopsis* sp. indicated that cells were 1.4-fold smaller under HL/HT (*t*-test, *t* = −4.57, df = 3.002, *P* < 0.05) despite no differences based on SSC (*t*-test, *t* = −0.0.244 df = 3.01, *P* = 0.8228). Accordingly, C and N cell contents were 1.2-fold and 1.4-fold lower under HL/HT, respectively (C cell content: *t*-test, *t* = −9.83, df = 24.50, *P* < 0.001. N cell content: *t*-test, *t* = −21.7, df = 24.50, *P* < 0.001). In addition, the Si cell content was unaffected by treatment (*t*-test, *t* = −0.761, df = 5.93, *P* = 0.476).

**Table II:** Cellular elemental content per species and treatment

Treatment	Cell size	C content	N content	Si content	Chl*a* content	FL3:SSC
	(μm)	(Pg cell^−1^)	(Pg cell^−1^)	(Pg cell^−1^)	(Pg cell^−1^)	(AU:AU)
*Fragilariopsis* sp*.*						
LL/LT	NA	4.1 ± 0.1	0.76 ± 0.01	0.001 ± 0.000	0.07 ± 0.00	9.59 ± 0.96
HL/HT	NA	3.5 ± 0.14	0.53 ± 0.02	0.001 ± 0.000	0.02 ± 0.01	4.31 ± 0.04
* P*-value		<0.001^*^	<0.001^*^	0.476	<0.001^*^	<0.005^*^
* t* value		−9.83	−21.70	−0.761	−16.22	−11.00
df		24.50	24.50	5.93	13.67	
*P. antarctica* SC						
LL/LT	5.7 ± 0.2	16.1 ± 1.5	NA	NA	NA	6.98 ± 0.57
HL/HT	6.0 ± 0.2	18.0 ± 1.3	NA	NA	NA	2.72 ± 0.24
* P*-value	0.268	0.172				<0.005^*^
* t* value	1.320	1.673				−11.93
df	3.43	3.89				
*P. antarctica* CC						
LL/LT	5.6 ± 0.5	15.8 ± 2.0	NA	NA	NA	NA
HL/HT	5.9 ± 0.4	17.1 ± 0.6	NA	NA	NA	NA
* P*-value	0.511	0.385				
* t* value	0.724	1.06				
df	3.80	2.35				

*t*-test was used for statistical analysis of differences between treatments, *P* < 0.05 are indicated with an asterisk (^*^). AU = arbitrary units. SC = single cells, CC = colonial cells.

In the case of *P. antarctica,* FL3:SSC for the single-cell fraction was 2.6-fold lower under HL/HT (*t*-test, *t* = −11.93, df = 2.70, *P* < 0.005). *Phaeocystis antarctica* did not show significant variation in cell size or C content on either of the morphotypes between treatments (Table II).

### Colony formation

The abundance of *P. antarctica* large colonies (≥20 μm) increased 6-fold under HL/HT (Table III). Similarly, the average diameter of colonies increased 4-fold under HL/HT (*t*-test, *t* = 4.06, df = 2.35, *P* < 0.05). Nevertheless, there were no significant differences in the total colony abundance per millilitre regardless of their size (*t*-test, *t* = 1.37, df = 3.13, *P* = 0.260). Results based on SFChl*a* revealed a 2-fold increase in the colony to single-celled biomass ratio (Ratio 20/02) at HL/HT (*t*-test, *t* = 14.37, df = 5.74, *P* < 0.001).

**Table III:** P. antarctica colony abundance and size

	Total colony abundance (colonies mL^−1^)	Abundance of colonies > 20-μm (colonies mL^−1^)	Ratio 20/02	Diameter (μm)	Min diameter (μm)	Max diameter (μm)
LL/LT	11.3 ± 3.7	2.2 ± 1.0	0.39 ± 0.03	20.8 ± 5.9	8.7	132.9
HL/HT	17.2 ± 6.6	12.9 ± 3.2	0.77 ± 0.04	73.2 ± 21.4	12.1	299.1
*P*-value	0.260	0.021^*^	<0.001^*^	0.042^*^		
*t* value	1.37	5.55	14.37	4.06		
df	3.13	2.38	5.74	2.35		

Values indicate the mean and SD. *t*-test was used for statistical analysis of differences between treatments; *P* < 0.05 are indicated with an asterisk (^*^).

### DMS and DMSPt concentrations

DMS and DMSPt concentrations increased under HL/HT for both species (Table IV). DMSPt:C and DMS:C increases of 1.5- and 1.7-fold occurred under HL/HT in *P. antarctica*, respectively, whereas *Fragilariopsis* sp. showed a fold increase of 1.3 on both parameters. DMSPt:Chl*a* and DMS:Chl*a* significantly increased 2.3- and 2.7-fold under HL/HT in *P. antarctica*, respectively, (DMSPt:Chl*a*; *t*-test, *t* = 12.39, df = 15.60, *P* < 0.001. DMS:Chl*a*; *t*-test, *t* = 13.08, df = 6.88, *P* < 0.001), while both variables showed a significant increase of 3.4-fold under HL/HT in *Fragilariopsis* sp. (DMSPt:Chl*a*; *t*-test *t* = 9.95, df = 5.27, *P* < 0.001. DMS:Chl*a*; *t* = 10.04, df = 5.13, *P* < 0.001). The DMSPt cell content in *Fragilariopsis* sp. was higher under HL/HT (*t*-test, *t* = 4.15, df = 7.21, *P* < 0.005), although the concentration was extremely low. There was no effect of treatment on DMSPt:DMS for either species (Table IV).

**Table IV:** DMSPt and DMS concentration and ratios for each species and treatment

Treatment	DMSPt:C	DMS:C	DMSPt:Chl*a*	DMS:Chl*a*	DMSPt:DMS	DMSPt content
	(mol:mol)	(mol:mol)	(mol:mol)	(mol:mol)	(mol:mol)	(pmol cell^−1^)
*Fragilariopsis* sp.						
LL/LT	0.003 ± 0.0003	1.53E-05 ± 3.80E-7	0.014 ± 0.001	0.003 ± 0.000	223 ± 19	0.0012 ± 0.0001
HL/HT	0.004 ± 0.0006	1.92E-05 ± 3.16E-6	0.047 ± 0.008	0.015 ± 0.003	216 ± 9	0.0014 ± 0.0000
* P*-value	0.015^*^	<0.001^*^	<0.001^*^	<0.001^*^	0.457	0.004^*^
* t* value	2.95	4.45	9.95	10.04	−0.79	4.15
df	9.67	11.34	5.27	5.13	7.15	7.21
*P. antarctica*						
LL/LT	0.006 ± 0.001	0.002 ± 0.000	0.042 ± 0.010	0.016 ± 0.003	2.6 ± 0.7	NA
HL/HT	0.009 ± 0.001	0.004 ± 0.000	0.096 ± 0.009	0.044 ± 0.005	2.2 ± 0.3	NA
* P*-value	<0.001^*^	<0.001^*^	<0.001^*^	<0.001^*^	0.166	
* t* value	5.43	8.11	12.39	13.08	−1.48	
df	12.72	8.76	15.60	6.88	11.33	

Values represent average and standard deviation. *t*-test was used for statistical analysis of differences between treatments; *P* < 0.05 are indicated with an asterisk (^*^). L/LT = low light/low temperature. HL/HT = high light/high temperature.

### Pigments, photoprotection and photoacclimation

**Fig. 3:**
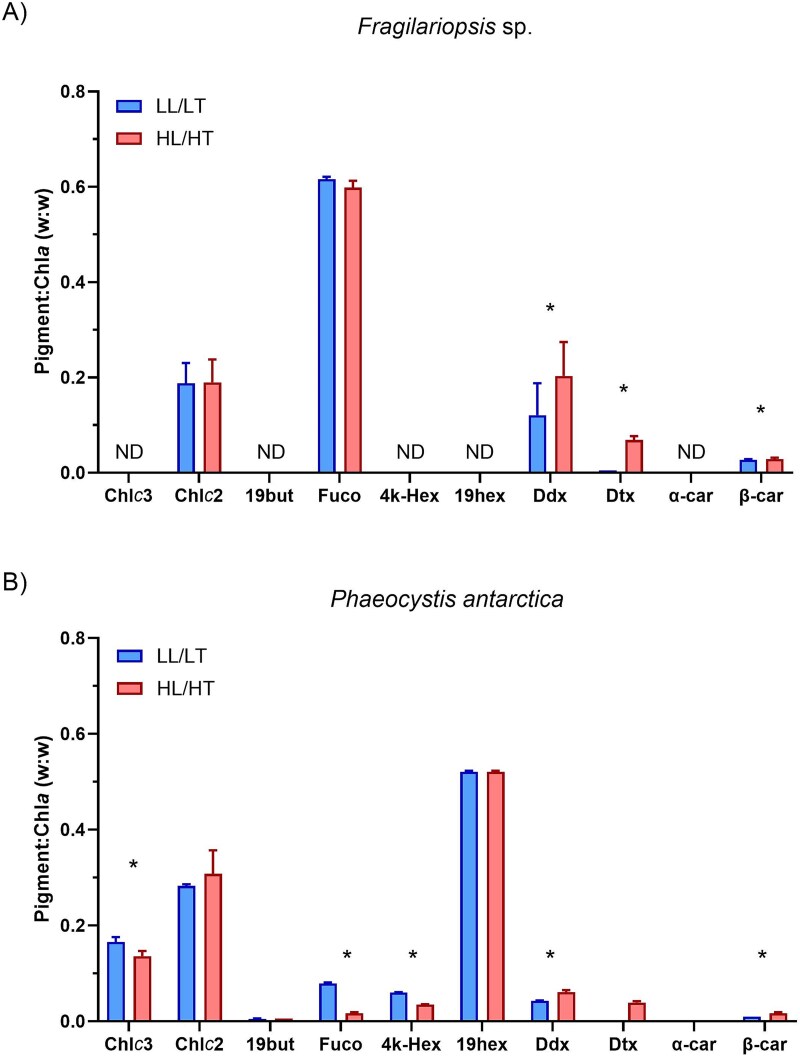
Pigment composition for each species and treatment. Mean (±SD) pigment to Chl*a* ratios (w:w) obtained from three replicated samples are shown. *t*-test was used for statistical analysis of differences between treatments; *P* < 0.05 are indicated with an asterisk (^*^). Values denoted as ND indicate that the pigment was not detected. Key: Chlorophyll *c*3 (Chl*c*3), Chlorophyll *c*2 (Chl*c*2), 19′butanoyloxyfucoxanthin (19but), Fucoxanthin (Fuco), 4keto-19′hexanoyloxyfucoxanthin (4 k-Hex), 19′hexanoyloxyfucoxanthin (19hex), Diadinoxanthin (Ddx), Diatoxanthin (Dtx), alpha-carotene (α-car) and beta-carotene (β-car). LL/LT = low light/low temperature. HL/HT = high light/high temperature. Values are available in Supplementary Table VI.

Xanthophyll cycle pigments increased under HL/HT for both species. Dtx in *Fragilariopsis* sp. was detectable in both treatments but only under HL/HT for *P. antarctica,* whereas Ddx was observed in both treatments for both species (Fig. 3). Dtx and Ddx increased 2.9-fold and 13.8-fold under HL/HT, respectively, in *Fragilariopsis* sp. In *P. antarctica,* Ddx increased 1.4-fold under HL/HT (Supplementary Table VI).

**Fig. 4:**
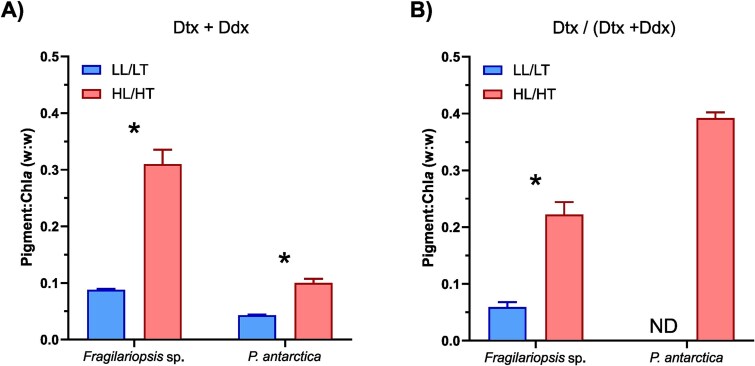
Pigments of the xanthophyll cycle. (**A**) For total xanthophyll pigments (Dtx + Ddx) relative to Chl*a*. (**B**) Ratio of de-epoxidation state (DES). The colour blue indicates low light/low temperature (LL/LT) and red high light/high temperature (HL/HT). *t*-test was used for statistical analysis of differences between treatments; *P* < 0.05 are indicated with an asterisk (^*^).

Total xanthophyll pigments (Dtx + Ddx) were 3.1-fold higher in *Fragilariopsis* sp. than *P. antarctica* under HL/HT (two-way ANOVA_,_  *F* = 115.6, *P* < 0.001, Fig. 4). However, the de-epoxidated state of the xanthophyll cycle (DES) was higher under HL/HT in *P. antarctica* (two-way ANOVA_,_  *F* = 265.1, *P* < 0.001). Other photoprotective pigments such as β-carotene (β-car) increased under HL/HT and had a higher concentration relative to TChl*a* in *Fragilariopsis* sp. (two-way ANOVA, *F* = 2.40, *P* < 0.001). In the case of primary pigment markers, fucoxanthin (Fuco) was at a higher concentration in *Fragilariopsis* sp. relative to *P. antarctica* (two-way ANOVA, *F* = 24.45, *P* < 0.001). Fuco was 4-fold lower under HL/HT in *P. antarctica* (*t*-test, *t* = −36.49, df = 3.4, *P* < 0.001) but did not show any difference between treatments in *Fragilariopsis* sp. (*t*-test, *t* = 0.26, df = 5.9, *P* = 0.806). 19′-hexanoyloxyfucoxanthin (19hex), 19′butanoyloxyfucoxanthin (19but) and 4keto-19′hexanoyloxyfucoxanthin (4 k-hex) were only detected in *P. antarctica* and were unaffected by treatment, except for a 1.7-fold decrease in 4 k-hex:Chl*a* under HL/HT (*t*-test, *t* = −30.88, df = 3.44, *P* < 0.0001). Chlorophyll-*c3* (Chl*c3*) was only detected in *P. antarctica* at a lower concentration under HL/HT (*t*-test, *t* = −3.44, df = 3.97, *P* < 0.05). Chlorophyll-*c2* (Chl*c2*) was identified in both species but with no significant treatment effect in either.

## DISCUSSION

This work measured acclimation responses for the colony-forming *P. antarctica* and *Fragilariopsis* sp. to a projected future scenario. The long exposure facilitated by the chemostat system ensured that the responses of both species were the result of acclimation instead of short-term adjustments (Ziv *et al.,* 2013). By using ecologically significant species and morphotypes (Smith *et al.,* 2003; Saggiomo *et al.,* 2021), the results provide ecologically relevant measurements relative to experiments using the single-celled morphotype of *P. antarctica* (Smith and Trimborn, 2024).

Results indicate that +2°C warming and increased light availability will result in physiological adjustment by both species with alterations in elemental composition, cell and colonial size as well as pigments. Elemental composition adjusted to high irradiance as expected (Geider, 1987), with a decrease in C:Chl*a* and FL3:SSC in both species. Although *Fragilariopsis* sp. had an expected photoacclimation response driven by a larger increase in C:Chl*a* and xanthophyll pigments, *P. antarctica* showed stronger activation of the xanthophyll cycle (1.6-fold higher), with a higher ratio of de-epoxidation state (DES) under HL/HT, than *Fragilariopsis* sp. (Fig. 4). A higher DES ratio in response to increasing in irradiance was anticipated for both species (Kropuenske *et al.,* 2009; Arrigo *et al.,* 2010), but the higher ratio in *P. antarctica* was surprising as this has not been described before. A prolonged DES in *Fragilariopsis* sp. is considered an additional photoprotective strategy for preventing damage in the event of re-exposure to high light after a drop in irradiance (Kropuenske *et al.,* 2009). In contrast, DES in *P. antarctica* has been proposed as a short-term photoprotective strategy to high light (Kropuenske *et al.,* 2009), which may reflect the different mixed layer depth preferences previously identified in these two species (Arrigo *et al.,* 1998). The long exposure to treatment in this experiment supports that this response is likely a long-term acclimation strategy in both species. This indicates that colony-forming *P. antarctica* can sustain the xanthophyll cycle for long periods and also potentially has greater reliance on the xanthophyll cycle than previously considered (Kropuenske *et al.,* 2009; Arrigo *et al.,* 2010).

In addition to pigments from the xanthophyll cycle, *P. antarctica* altered its pigment composition to a greater extent than *Fragilariopsis* sp. under HL/HT, with significant decreases in Chlorophyll-*c*3, Fucoxanthin and 4keto-19′hexanoyloxyfucoxanthin (Fig. 3). The decrease of these pigments under HL/HT is consistent with their role as light-harvesting pigments (Van Leeuwe *et al.,* 2014) and shows a wider pigment-driven acclamatory response for *P. antarctica* relative to *Fragilariopsis* sp*.,* whose main alterations were in C:Chl*a* and total xanthophyll pigments. The lack of treatment effect on the two main taxonomic pigment markers—fucoxanthin in *Fragilariopsis* sp*.* and 19′-hexanoyloxyfucoxanthin in *P. antarctica—*shows a tight balance of these pigments with Chl*a* concentration, a response that has been previously described for *P. antarctica* (Van Leeuwe *et al.,* 2014), supporting the use of these pigments for taxonomic identification.

Acclimatory responses to warming and elevated light also included a reduction in the cell size in *Fragilariopsis* sp. (1.3-fold smaller) and an increase in the colonial size and abundance in *P. antarctica* (4-fold increase). The observed alterations in cell size and photophysiology in response to future projections could alter the carbon export and transfer to higher trophic levels. Initial assessment of carbon export by *P. antarctica* suggested a high export potential through sinking for this species (DiTullio *et al.,* 2000), favoured to some extent by the lower grazing susceptibility of larger colonies (Tang, 2003). *Phaeocystis antarctica* colony size increased under HL/HT, in agreement with previous work evaluating the effect of irradiance in colony formation (Peperzak, 1993) and also experiments using natural communities (Feng *et al.,* 2010), which suggests increased protection from grazing (Tang, 2003). However, this size increase will not necessarily enhance carbon export, as a significant proportion of *P. antarctica* carbon is remineralized in surface layers by bacterial activity (Thingstad and Billen, 1994) including colonies that sink quickly (Asper and Smith, 2019). Furthermore, bacterial remineralization may be further enhanced under higher temperatures (Segschneider and Bendtsen, 2013). Overall, this suggests a low carbon export efficiency by *P. antarctica* (Meyer *et al.,* 2022). Conversely, the smaller but denser silica frustules in *Fragilariopsis* sp. under the HL/HT treatment could enhance diatom-driven carbon export in future warmer conditions by increasing their specific sinking rates (Lürling, 2021; Raven and Waite, 2004; Brzezinski *et al*., 2015). Denser cells would also provide an ecological advantage by greater protection against viral infection (Kranzler *et al*., 2019) and grazing (Pančić *et al*., 2019). Enhanced sinking rates and a reduction in cell loss to viral lysis and grazing could lead to more efficient export of both carbon and silica. Remineralization rates differ, with silica being slower than carbon (Nelson *et al.,* 1996) and strongly dependent on temperature (Lawson *et al.,* 1978,), and so evaluating the combined effect of higher temperatures and light is needed to estimate the future contribution to silica fluxes.

Sulphur cycling as indicated by DMS and DMSPt was promoted by increasing temperature and light. Although the Southern Ocean is projected to decrease the DMS ocean surface concentration (Kloster *et al.,* 2007), the trend for regions where these two species dominate, like the Ross Sea, is to increase in response to retreating sea ice, enhancing light availability and increasing primary productivity (Kloster *et al.,* 2007), in agreement with the scenario tested in this work. Previous studies suggest that higher irradiance may promote DMS and DMSP concentrations by increasing biomass and also by enhancing cellular release of these compounds (Archer *et al.,* 2010). Here, the lack of treatment effect on DMSPt:DMS ratios suggests that pathways involved in the cleavage of DMSP to DMS were unaffected, reflecting a balance between the metabolism of DMSP and the DMSP lyase activity. Higher DMSP in response to HL/HT supports its role as an antioxidant (Sunda *et al.,* 2002) and suggests an important physiological role in photoprotection on both species. On the other hand, an increase in DMS could further impact grazing dynamics due to its role as an info-chemical with an important ecological influence (Garcés *et al.,* 2013). Description of this compound as a grazing attractor (Shemi *et al.,* 2021) suggests that the protection conceived by increases in colony size (*P. antarctica*) may be offset, especially in future shallower surface mixed layers where the predator–prey encounter rate is expected to be higher (Behrenfeld, 2010).

Competition between *Fragilariopsis* sp. and *P. antarctica* will influence the regional relative fate of carbon between export and the food web. Previous experiments have determined that *Fragilariopsis* sp*.* shows a greater capacity to thrive under stratified conditions (Xu *et al.,* 2014), while others have reported an increase in the colonial and relative abundance of *P. antarctica* relative to diatoms under an HL scenario (Feng *et al.,* 2010). However, the combination of high DES observed here in *P. antarctica* with the reported repair mechanism in response to high irradiance (Kropuenske *et al.,* 2009) may support adaptation to elevated irradiance, consistent with the observed dominance of this species under stratified conditions (Mangoni *et al.,* 2019; Cristi *et al.,* 2024). This unexpectedly physiological capability of *P. antarctica* reinforces the high genetic plasticity of *P. antarctica,* with blooms presenting clones that respond differently to the environment (Luxem *et al.,* 2017) and indicates that *P. antarctica* can be a better competitor in a future scenario than anticipated. This high genotypic diversity emphasises the value of experiments to expand the understanding of traits for key species under different environmental conditions.

## CONCLUSIONS

Differential changes in *Fragilariopsis* sp. and *P. antarctica* elemental and cellular stoichiometry were observed in response to higher irradiance and temperature. The stronger DES observed in *P. antarctica* indicates that the colony-forming strain possesses mechanisms for tolerance to the higher irradiance experienced in a shallow mixed layer depth, contrary to previous observations. This pigment response was coupled with an increase in colony size and elevated DMS(P):C, which may have ecological implications related to grazing and potentially vertical export under future conditions. These results provide valuable insights for trophic and biogeochemical models aiming to predict changes in the spatial and temporal distribution of phytoplankton community composition in the Southern Ocean, including the Ross Sea, where these two species tend to dominate. Overall, the results indicate that *P. antarctica* has the potential to compete with diatoms under warmer high irradiance conditions in Southern ocean surface waters. Further multifactorial experiments, including the combination of HL/LT and LL/HT with iron control of growth rates should be conducted to further understand the effect of these climate-sensitive variables on the physiology of both taxa. Including the effect of changing environmental conditions on associated bacteria as well as in grazing behaviour would be necessary to determine the impacts of changing bloom dynamics in carbon flows.

## Supplementary Material

Supplementary_material_fbaf023

## Data Availability

Data are available at https://github.com/apcristi/Chemostat-data.
