# Supplementary material for: Eco-physiological response of Phaeocystis antarctica and Fragilariopsis sp. to increases in irradiance and temperature
Source: J Plankton Res. 2025 Jun 15;47(4):fbaf023. doi: 10.1093/plankt/fbaf023 (PMC12167505; doi:10.1093/plankt/fbaf023)
Supplement: Supplementary_material_fbaf023 [file supplementary_material_fbaf023.docx]

**Supplementary material for:** **Eco-physiological response of *Phaeocystis antarctica* and *Fragilariopsis* sp. to increases in irradiance and temperature projected for the Ross Sea**

Antonia Cristi^1, 2^, Stacy Deppeler^1^, Alexia Saint-Macary^1,2^, Andrew Marriner^1^, Mikel Latassa^3^, Cliff S. Law^1,2^, and Andrés Gutiérrez-Rodríguez^1,3^

1.- National Institute of Water and Atmospheric Research, Wellington, New Zealand

2.- Department of Marine Sciences, University of Otago, Dunedin, New Zealand

3.- Instituto Español de Oceanografía, Centro Oceanográfico de Gijón/ Xixón (IEO, CSIC), Asturias, España


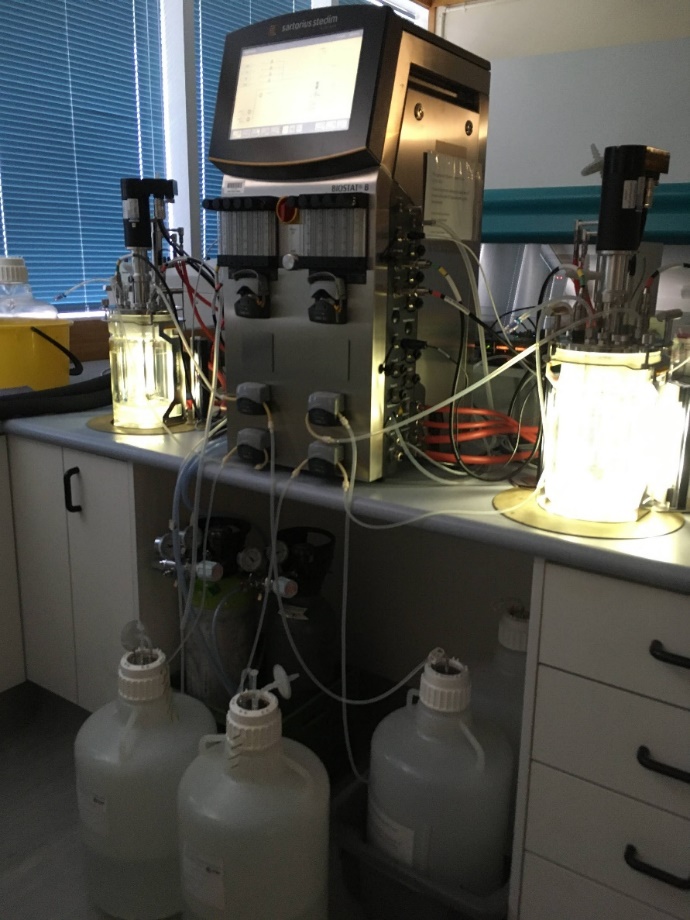


**Supplementary Fig.1.** Chemostat set-up. Reflective screens have been removed in this image.

**S1.1 Biostat equipment set up**

The Biostat experimental system was set up as follows:

Each Univessel® Glass 2L culture vessel (the “chamber”) contained an internal motorised stainless-steel stirrer, level control sensor, temperature probe in stainless-steel pocket, air exhaust outlet with PTFE filter, multiple stainless-steel dip tubes, and media inlet and outlets. The chambers were initially set up with all sensors and tubes connected and autoclaved with 250 ml distilled water inside to sterilise all internal components. After cooling, the distilled water was removed aseptically by a syringe using a sterile tube attached to the chamber. The temperature probe was placed into the stainless-steel pocket after sterilising.

The chambers and sensors were connected to each side of the Biostat B control tower. Sterile media was pumped into each chamber by an integrated peristaltic pump, with the level sensor set to the appropriate volume required. Media intake was controlled by a continuously running integrated peristaltic pump, connected to a sterile media carboy and set at a designated speed for each specific culture. Media removal was activated by the level sensor, which triggered a second integrated peristaltic pump, removing excess media to a waste carboy.

Lighting was provided by four 6000K, 20W pure white LED lights fitted to two brackets placed at the back of the chamber on the left and right sides. A screen covered with reflective material was placed on the opposite side of the chamber to reflect light back into the chamber and block out external light sources. The light intensity was measured before setting up the chambers with a Biospeherical Instruments Quantum Scalar Irradiance PAR sensor (QSL-2100), placed into the centre of each Univessel culture vessel filled with filtered seawater. Light intensity was set by adjusting the voltage of the LED lights.

Temperature control was achieved using a recirculating chiller (Frigomix 1000, Sartorius stedim biotech, Göttingen, Germany) filled with 25% glycol solution, with individual chamber temperature set and controlled by the Biostat operation software and monitored by the *in situ* temperature probes.

All sampling was performed aseptically from a sterile sampling tube with a luer-activated access site attached to a dip tube in the culture vessel (Benchmark™ sampling line, Sartorius stedim biotech, Göttingen, Germany).

**Supplementary Table I.** Blastn analysis for the *Fragilariopsis* strain RCC6062. The table shows the best 10 matches for the query ID: [MK542687.1](https://www.ncbi.nlm.nih.gov/sites/entrez?cmd=Search&db=nucleotide&term=MK542687.1&dopt=GenBank)

| Scientific Name | Max Score | Total Score | Query Cover | E value | Per. ident | Acc. Len | Accession |
| --- | --- | --- | --- | --- | --- | --- | --- |
| Fragilariopsis sp. | 1858 | 1858 | 100% | 0 | 100 | 1677 | [MN824020.1](https://www.ncbi.nlm.nih.gov/nucleotide/MN824020.1?report=genbank&log$=nucltop&blast_rank=1&RID=K2YH0EA6016) |
| Fragilariopsis sp. | 1858 | 1858 | 100% | 0 | 100 | 1670 | [MN824018.1](https://www.ncbi.nlm.nih.gov/nucleotide/MN824018.1?report=genbank&log$=nucltop&blast_rank=2&RID=K2YH0EA6016) |
| Fragilariopsis sp. | 1858 | 1858 | 100% | 0 | 100 | 1608 | [MN824014.1](https://www.ncbi.nlm.nih.gov/nucleotide/MN824014.1?report=genbank&log$=nucltop&blast_rank=3&RID=K2YH0EA6016) |
| Fragilariopsis sp. | 1858 | 1858 | 100% | 0 | 100 | 1633 | [MN824010.1](https://www.ncbi.nlm.nih.gov/nucleotide/MN824010.1?report=genbank&log$=nucltop&blast_rank=4&RID=K2YH0EA6016) |
| Fragilariopsis sp. | 1858 | 1858 | 100% | 0 | 100 | 1658 | [MN824005.1](https://www.ncbi.nlm.nih.gov/nucleotide/MN824005.1?report=genbank&log$=nucltop&blast_rank=5&RID=K2YH0EA6016) |
| Fragilariopsis sp. | 1858 | 1858 | 100% | 0 | 100 | 1674 | [MN824004.1](https://www.ncbi.nlm.nih.gov/nucleotide/MN824004.1?report=genbank&log$=nucltop&blast_rank=6&RID=K2YH0EA6016) |
| Fragilariopsis sp. RCC6073 | 1858 | 1858 | 100% | 0 | 100 | 1084 | [MK542698.1](https://www.ncbi.nlm.nih.gov/nucleotide/MK542698.1?report=genbank&log$=nucltop&blast_rank=7&RID=K2YH0EA6016) |
| Fragilariopsis sp. RCC6062 | 1858 | 1858 | 100% | 0 | 100 | 1030 | [MK542687.1](https://www.ncbi.nlm.nih.gov/nucleotide/MK542687.1?report=genbank&log$=nucltop&blast_rank=8&RID=K2YH0EA6016) |
| Fragilariopsis cylindrus | 1858 | 1858 | 100% | 0 | 100 | 1747 | [LC189084.1](https://www.ncbi.nlm.nih.gov/nucleotide/LC189084.1?report=genbank&log$=nucltop&blast_rank=9&RID=K2YH0EA6016) |
| Fragilariopsis cylindrus | 1858 | 1858 | 100% | 0 | 100 | 1778 | [ON888448.1](https://www.ncbi.nlm.nih.gov/nucleotide/ON888448.1?report=genbank&log$=nucltop&blast_rank=10&RID=K2YH0EA6016) |

.


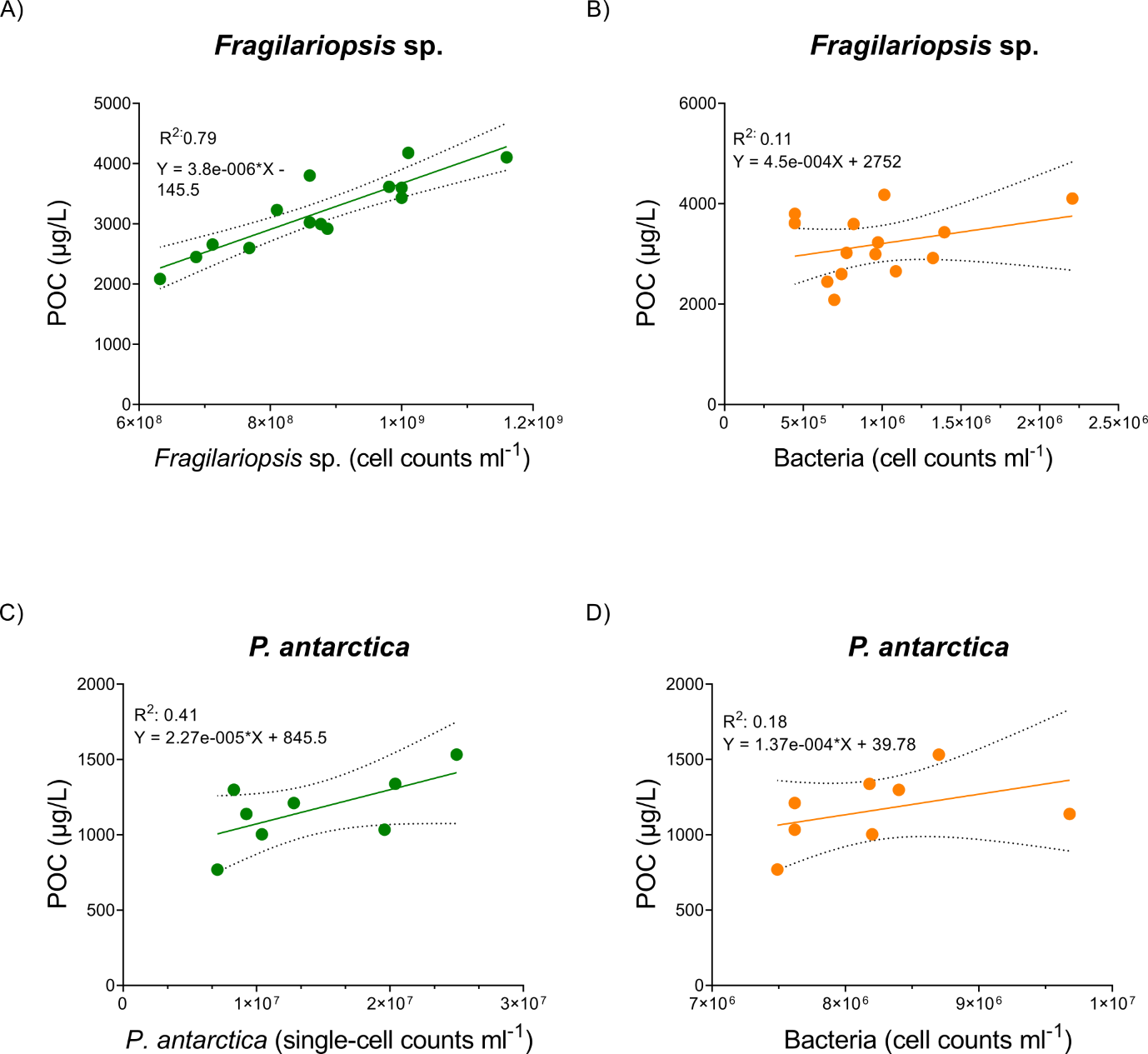


**Supplementary Fig.2.** Correlation between POC concentration and cell counts during the progression of the experiment. A) and B) for *Fragilariopsis* sp. C) and D) for *P. antarctica*. The figure on the left (in green) indicates the correlation between POC concentration and phytoplankton cell counts, whereas the figure on the right (in orange) shows the correlation between bacterial counts and POC.

**Supplementary Table II**. Flow Cytometry measurements per species and treatment. Fra = *Fragilariopsis* sp., PA = *Phaeocystis antarctica*. SSC= Side scatter, FSC=Forward scatter, FL3= Chla fluorescence. LL/LT = Low light/Low Temperature. HL/HT = High-light/High-Temperature. SSC, FSC and FL3 are in arbitrary units (AU). Note that PA only includes single cells.

| Experiment | Treatment | Replicate | Mean SSC-H | Mean FSC-H | Mean FL3-H | FL3/SSC | CV SSC-H | CV FSC-H | CV FL3-H | Eukaryotic Nanophytoplankton 2-20 um Cells mL^-1^ |
| --- | --- | --- | --- | --- | --- | --- | --- | --- | --- | --- |
| Fra | LL/LT | R1 | 31256.73 | 479373.81 | 325399.44 | 10.41 | 1.35 | 0.38 | 0.45 | 1026130 |
| Fra | LL/LT | R2 | 35125.00 | 484473.25 | 333653.28 | 9.50 | 1.65 | 0.38 | 0.46 | 1028410 |
| Fra | LL/LT | R3 | 46592.88 | 602030.88 | 385150.91 | 8.27 | 1.11 | 0.31 | 0.37 | 933940 |
| Fra | LL/LT | R4 | 30510.94 | 479167.69 | 310195.75 | 10.17 | 0.96 | 0.37 | 0.43 | 1043970 |
| Fra | HL/HT | R1 | 35147.58 | 372695.38 | 150157.63 | 4.27 | 0.89 | 0.44 | 0.52 | 1171230 |
| Fra | HL/HT | R2 | 34901.55 | 374183.22 | 150825.20 | 4.32 | 0.89 | 0.46 | 0.55 | 1155610 |
| Fra | HL/HT | R3 | 34652.56 | 371549.50 | 150898.77 | 4.35 | 0.86 | 0.45 | 0.54 | 1166970 |
| Fra | HL/HT | R4 | 35153.34 | 373281.50 | 150724.20 | 4.29 | 0.88 | 0.45 | 0.55 | 1164220 |
| PA | LL/LT | R1 | 80395.32 | 614633.44 | 537113.06 | 6.68 | 1.30 | 0.60 | 0.84 | 11952 |
| PA | LL/LT | R2 | 75463.63 | 628578.63 | 500486.09 | 6.63 | 0.99 | 0.59 | 0.84 | 12144 |
| PA | LL/LT | R3 | 70724.88 | 614346.94 | 540063.25 | 7.64 | 0.91 | 0.60 | 0.82 | 14452 |
| PA | HL/HT | R1 | 107049.32 | 715271.38 | 280685.25 | 2.62 | 1.03 | 0.65 | 1.07 | 35028 |
| PA | HL/HT | R2 | 105778.66 | 725745.25 | 269512.81 | 2.55 | 1.18 | 0.63 | 1.07 | 22340 |
| PA | HL/HT | R3 | 103763.15 | 804493.88 | 311068.06 | 3.00 | 0.90 | 0.58 | 0.98 | 17760 |

**Supplementary Table III.** Two-way ANOVA between species and experimental conditions. “Fraction” in C cell^-1^ *P. antarctica* refers to single-cell vs colony-forming cells. Variables with *p*<0.05 are indicated with an asterisk (*).

|  |  | Df | Sum Sq | Mean Sq | F | P value | Pr(>F) |
| --- | --- | --- | --- | --- | --- | --- | --- |
| C:N | Treatment | 1 | 12.17 | 12.17 | 194.45 | < 2e-16 | * |
|  | Species | 1 | 25.85 | 25.85 | 413.19 | < 2e-16 | * |
|  | Treatment:Species | 1 | 0.63 | 0.63 | 10.13 | 0.003 | * |
| C:Chla | Treatment | 1 | 50005 | 50005 | 299.59 | < 2e-16 | * |
|  | Species | 1 | 6138 | 6138 | 36.78 | 4.7E-07 | * |
|  | Treatment:Species | 1 | 1833 | 1833 | 10.98 | 0.002 | * |
| DMSPt:C | Treatment | 1 | 3.0E-05 | 3.0E-05 | 37.58 | 1E-06 | * |
|  | Species | 1 | 1.0E-04 | 1.0E-04 | 131.65 | 2E-12 | * |
|  | Treatment:Species | 1 | 9.6E-06 | 9.6E-06 | 12.18 | 2E-03 | * |
| DMS:C | Treatment | 1 | 1.21E-06 | 1.21E-06 | 23 | 3E-05 | * |
|  | Species | 1 | 8.41E-05 | 8.41E-05 | 1597 | 2E-16 | * |
|  | Treatment:Species | 1 | 6.52E-06 | 6.52E-06 | 123.8 | 5E-13 | * |
| Fuco | Treatment | 1 | 0.002 | 0.002 | 14.8 | 0.003 | * |
|  | Species | 1 | 1.073 | 1.073 | 7091.99 | 0.000 | * |
|  | Treatment:Species | 1 | 0.004 | 0.004 | 24.45 | 0.001 | * |
| C cell^-1^  *P. antarctica* | Treatment | 1 | 7.62 | 7.62 | 3.69 | 0.091 |  |
|  | Fraction | 1 | 1.10 | 1.10 | 0.54 | 0.485 |  |
|  | Treatment:Fraction | 1 | 0.30 | 0.30 | 0.15 | 0.712 |  |
| DMSPt:Chla | Treatment | 1 | 0.02 | 0.02 | 237.69 | 1E-14 | * |
|  | Species | 1 | 0.01 | 0.01 | 160.07 | 1E-12 | * |
|  | Treatment:Species | 1 | 0.00 | 0.00 | 13.19 | 1E-03 | * |
| DMS:Chla | Treatment | 1 | 0.00 | 0.00 | 357.21 | < 2e-16 | * |
|  | Species | 1 | 0.00 | 0.00 | 356.85 | < 2e-16 | * |
|  | Treatment:Species | 1 | 0.00 | 0.00 | 52.31 | 1E-07 | * |

**Supplementary Table IV.** Tukey's HSD test on parameter response for both species. Variables with *p*<0.05 are indicated with an asterisk (*). PA = *Phaeocystis antarctica*. Fra = *Fragilariopsis* sp., Fuco = Fucoxanthin, HL/HT = High-Light/High-Temperature, LL/LT = Low-Light/Low-Temperature.

|  | | Diff | lwt | upr | P adj |  |
| --- | --- | --- | --- | --- | --- | --- |
| C:N | | | | | | |
| HL/HT PA | HL/HT Fra | -1.733 | -2.010 | -1.455 | 0.00 | * |
| LL/LT PA | HL/HT Fra | -2.419 | -2.697 | -2.141 | 0.00 | * |
| HL/HT PA | LL/LT Fra | -0.577 | -0.855 | -0.299 | 0.00 | * |
| LL/LT PA | LL/LT Fra | -1.263 | -1.541 | -0.986 | 0.00 | * |
| C:Chla | | | | | | |
| HL/HT PA | HL/HT Fra | 11.081 | -4.224 | 26.385 | 0.23 |  |
| LL/LT PA | HL/HT Fra | -42.674 | -57.979 | -27.370 | 0.00 | * |
| HL/HT PA | LL/LT Fra | 91.532 | 76.228 | 106.837 | 0.00 | * |
| LL/LT PA | LL/LT Fra | 37.777 | 22.473 | 53.082 | 0.00 | * |
| Fuco | | | | | | |
| HL/HT PA | HL/HT Fra | -0.592 | -0.621 | -0.564 | 0.00 | * |
| LL/LT PA | HL/HT Fra | -0.529 | -0.558 | -0.501 | 0.00 | * |
| HL/HT PA | LL/LT Fra | -0.589 | -0.618 | -0.561 | 0.00 | * |
| LL/LT PA | LL/LT Fra | -0.527 | -0.555 | -0.498 | 0.00 | * |

**Supplementary Table V.** Stoichiometric ratios for cellular composition for both species and treatments. *t*-test was used for statistical analysis of differences between treatments, *p*<0.05 are indicated with an asterisk (*). HL/HT = High-Light/High-Temperature, LL/LT = Low-Light/Low-Temperature.

| Treatment | C:N  (w:w) | C:Chl*a*  (w:w) | Si:C  (mol:mol) | Si:N  (mol:mol) |
| --- | --- | --- | --- | --- |
| *Fragilariopsis* sp. |  |  |  |  |
| LL/LT | 5.4 ± 0.1 | 56 ± 2 | 0.05 ± 0.00 | 0.30 ± 0.01 |
| HL/HT | 6.6 ± 0.4 | 136 ± 13 | 0.05 ± 0.01 | 0.42 ± 0.03 |
| *P*-value  *t* value  df | <0.001*  11.94  18.23 | <0.001*  12.33  11.20 | 0.401  0.862  17.27 | <0.001*  18.89  21.45 |
| *P. antarctica* |  |  |  |  |
| LL/LT | 4.2 ± 0.2 | 94 ± 6 | NA | NA |
| HL/HT | 4.9 ± 0.2 | 148 ± 8 | NA | NA |
| *P*-value  *t* value  df | <0.001*  7.34  15.87 | <0.001*  16.95  14.51 | NA | NA |


**Supplementary Table VI**- Pigment Composition. Mean (± SD) pigment to Chla ratios (w:w) obtained from three replicated samples are shown. t-test was used for statistical analysis of differences between treatments, *p*<0.05 are indicated with an asterisk (*). Values denoted as ND indicate that the pigment was not detected. Key: Chlorophyll c3 (Chlc3), Chlorophyll c2 (Chlc2), 19’butanoyloxyfucoxanthin (19but), Fucoxanthin (Fuco), 4keto-19'hexanoyloxyfucoxanthin (4k-Hex), 19'hexanoyloxyfucoxanthin (19hex), Diatoxanthin (Dtx), Diadinoxanthin (Ddx), de-epoxidate state of the xanthophyll cycle (DES), alpha-carotene (α-car) and beta-carotene (β-car). HL/HT = High-Light/High-Temperature, LL/LT = Low-Light/Low-Temperature.

| Treatment | Chl*c3* | Chl*c2* | 19but | Fuco | 4k-Hex | 19hex | Dtx | Ddx | DES | α-car | β-car |
| --- | --- | --- | --- | --- | --- | --- | --- | --- | --- | --- | --- |
| *Fragilariopsis* sp. |  |  |  |  |  |  |  |  |  |  |  |
| LL/LT | ND | 0.166±0.060 | ND | 0.606±0.01 | ND | ND | 0.005±0.01 | 0.083±0.001 | 0.059±0.009 | ND | 0.026±0.001 |
| HL/HT | ND | 0.212±0.021 | ND | 0.609±0.067 | ND | ND | 0.069±0.08 | 0.241±0.022 | 0.222±0.022 | ND | 0.030±0.002 |
| *P*-value  *t* value  df | NA | 0.220  1.47  3.73 | NA | 0.806  0.26  5.99 | NA | NA | <0.001*  16.02  3.06 | <0.001*  14.70  3.01 | <0.001*  14.06  3.92 | NA | 0.031*  3.31  3.88 |
| *P. antarctica* |  |  |  |  |  |  |  |  |  |  |  |
| LL/LT | 0.166±0.010 | 0.283±0.001 | 0.005±0.001 | 0.079±0.002 | 0.060±0.001 | 0.521±0.002 | ND | 0.043±0.001 | ND | 0.002±0.000 | 0.010±0.000 |
| HL/HT | 0.157±0.018 | 0.308±0.049 | 0.006±0.002 | 0.017±0.002 | 0.035±0.001 | 0.521±0.002 | 0.039±0.003 | 0.061±0.004 | 0.392±0.010 | ND | 0.017±0.002 |
| *P*-value  *t* value  df | 0.026*  -3.44  3.97 | 0.465  0.89  2.01 | 0.084  2.73  2.59 | <0.001*  -36.49  3.40 | <0.001*  -30.88  3.44 | 0.999  0.003  2.92 | NA | 0.012*  7.05  2.35 | NA | NA | 0.021*  6.43  2.08 |
